# Nonalcoholic Fatty Liver: A Possible New Target for Type 2 Diabetes Prevention and Treatment

**DOI:** 10.3390/ijms141122933

**Published:** 2013-11-20

**Authors:** Barbara Fruci, Stefania Giuliano, Angela Mazza, Roberta Malaguarnera, Antonino Belfiore

**Affiliations:** Endocrinology, Department of Health Sciences, University Magna Graecia of Catanzaro, Catanzaro 88100, Italy; E-Mails: barbara.fruci@yahoo.it (B.F.); stefania.giuliano75@gmail.it (S.G.); angela_mazza@libero.it (A.M.); robertamlg@hotmail.com (R.M.)

**Keywords:** nonalcoholic fatty liver disease (NAFLD), insulin resistance, type 2 diabetes mellitus (T2DM), cardiovascular disease (CVD), antidiabetic drugs, statins

## Abstract

Non-alcoholic fatty liver disease (NAFLD) is the most common liver disorder worldwide. Several lines of evidence have indicated a pathogenic role of insulin resistance, and a strong association with type 2 diabetes (T2MD) and metabolic syndrome. Importantly, NAFLD appears to enhance the risk for T2MD, as well as worsen glycemic control and cardiovascular disease in diabetic patients. In turn, T2MD may promote NAFLD progression. The opportunity to take into account NAFLD in T2MD prevention and care has stimulated several clinical studies in which antidiabetic drugs, such as metformin, thiazolidinediones, GLP-1 analogues and DPP-4 inhibitors have been evaluated in NAFLD patients. In this review, we provide an overview of preclinical and clinical evidences on the possible efficacy of antidiabetic drugs in NAFLD treatment. Overall, available data suggest that metformin has beneficial effects on body weight reduction and metabolic parameters, with uncertain effects on liver histology, while pioglitazone may improve liver histology. Few data, mostly preclinical, are available on DPP4 inhibitors and GLP-1 analogues. The heterogeneity of these studies and the small number of patients do not allow for firm conclusions about treatment guidelines, and further randomized, controlled studies are needed.

## 1. Introduction

Hepatic steatosis represents the first step of non-alcoholic fatty liver disease (NAFLD). The spectrum of this disease ranges from simple steatosis to non-alcoholic steatohepatitis (NASH), fibrosis, cirrhosis and hepatocellular carcinoma. NAFLD is considered the most common cause of chronic liver disease worldwide [1,2]. In the general US population, the prevalence of NAFLD is estimated to be approximately 30% [3], but much higher estimates are reported in selected high-risk populations, such as Hispanics, obese persons, and patients with type 2 diabetes mellitus (T2DM) or with metabolic syndrome [2]. Several lines of evidence suggest that NAFLD promotes T2DM, and it is an independent determinant of cardiovascular disease (CVD) [4]. NAFLD is, therefore, a complex disease with clinical and therapeutic implications beyond the liver disease. In the light of the strong link between NAFLD and T2DM, our review discusses the possible role of antidiabetic drugs in NAFLD therapy, and underlines the need to take into account NAFLD in diabetes prevention and care.

## 2. NAFLD Is Associated with Insulin Resistance

NAFLD is primarily characterized by accumulation of triacylglycerol in the liver [5]. This condition is due to an imbalance in any of the pathways involved in triacylglycerol delivery, synthesis, export or oxidation. Fatty acids come to the liver from different sources: from diet, and from adipocytes via lipolysis and *de novo* lipogenesis [6].

Epidemiological studies [7–14] clearly show a very high prevalence of NAFLD in conditions associated with insulin resistance, such as obesity, T2DM and metabolic syndrome. While NAFLD is present in 20%–30% of the general population [9], it reaches the impressive prevalence of 75% and 90% in obese [8,13,14] and morbidly obese patients [10,11], respectively. NAFLD is also present in a high proportion (ranging 50%–75%) of patients affected by T2DM [7,12], and is so strongly associated with metabolic syndrome [7,12] that it is often considered the hepatic component of metabolic syndrome [15].

Insulin resistance is believed to represent a common pathogenic factor underlying NAFLD and these metabolic disorders [16]. In fact, NAFLD is strongly associated with insulin resistance, not only at the level of liver but also at the level of muscle and adipose tissue. A number of studies [17–19] conducted in NAFLD patients have shown both an impaired ability of insulin to suppress endogenous glucose production, indicating the presence of hepatic insulin resistance, and an approximately 50% reduction in glucose disposal, a measure of whole-body insulin sensitivity. Moreover, NAFLD patients show a reduced insulin-mediated inhibition of lipolysis [20–22], that results in increased flux of free fatty acids (FFAs) to the liver and in a blunted inhibition of fatty acid oxidation. This mechanism reflects the decreased uptake and use of glucose as a source of energy [18]. Excess caloric intake contributes to fatty liver directly by providing an excess of dietary fat, and indirectly by favoring obesity and, therefore, insulin resistance. The increased amount of adipose tissue provides a major source of FFAs. Insulin resistance increases the FFAs flux from the adipocytes to the liver because of the reduced ability of insulin in inhibiting lipolysis. Furthermore, obesity worsens liver fat accumulation indirectly, through a reduced production of adiponectin in the adipose tissue that results in a decreased fatty acid oxidation in the liver.

## 3. NAFLD, Consequence or Cause of Insulin Resistance

NAFLD is strictly associated with insulin resistance. However, whether NAFLD is a consequence or a cause of insulin resistance is a matter of debate.

### 3.1. NAFLD: Consequence of Insulin Resistance

Several animal models support a direct causal relationship between insulin resistance, compensatory hyperinsulinemia and hepatic steatosis [23]. Genetically modified NAFLD mice, such as SREBP-1c transgenic mice, ob/ob and db/db mice, are characterized by insulin resistance. Ota *et al*. [24] investigated the role of insulin resistance in the development of NAFLD/NASH. They studied methionine and choline-deficient (MCD) diet-induced NASH in rats with obesity/diabetes background and fed with a high fat (HF) diet to induce insulin resistance. Their results showed that obesity, diabetes and HF diet accelerated not only steatosis but also inflammation and fibrosis in the liver, supporting a causal role of insulin resistance in the development/progression of NAFLD. Moreover, they demonstrated a beneficial effect of pioglitazone—a drug that improves insulin resistance—on steatohepatitis pathology in this model.

Studies in patients with metastatic insulin-secreting tumors (insulinomas) or with pancreatic islet cell transplants provide further evidence that insulin directly promotes fat accumulation in liver cells. Hepatocytes surrounding metastatic insulinomas become engorged with triglycerides, as do hepatocytes surrounding transplanted islet cells [25].

More evidence that insulin resistance causes steatosis derives from patients with AKT2 mutations [26]. In this pathological condition, patients show profound resistance to the glucoregulatory actions of insulin but retain sensitivity to the lipogenic effects of the hormone.

In normal liver, insulin signaling inhibits glucose production and promotes fatty acid synthesis [27]. In contrast, in hyperinsulinemic subjects the inhibitory effect of insulin on glucose production is diminished, whereas the stimulatory effect of insulin on liver lipogenesis is retained or increased [28]. In fact, hyperinsulinemia activates the transcriptional factor SREBP-1 (sterol receptor binding protein 1-c) promoting lipogenic enzyme gene expression in spite of insulin resistance [29]. Furthermore, high levels of insulinemia may also contribute to triacylglycerol accumulation in the liver through the suppression of Foxa2 transcription factor, which promotes fatty acid oxidation [30]. Hyperglycemia can also stimulate lipogenesis by activating the carbohydrate response element binding protein (ChREBP) resulting in the transcription of genes involved in glycolysis and lipogenesis [31].

### 3.2. NAFLD: Cause of Insulin Resistance

The notion that excess triacylglycerol in liver causes insulin resistance is actually more debated. Evidences that liver steatosis may cause development of insulin resistance come from certain animal models [23,32].

For instance, mice with targeted overexpression of lipoprotein lipase (LPL) in the liver develop liver-specific steatosis associated with liver-specific hepatic insulin resistance [33]. Rats with high-fat diet induced hepatic steatosis undergo hepatic insulin resistance before obesity develops and circulating adipocytokines increase [34]. Further evidence supporting the role of intrahepatic lipid accumulation in mediating hepatic insulin resistance comes from the treatment of high-fat diet rats with low doses of 2,4-dinitrophenol (DNP). DNP, by promoting mitochondrial energy uncoupling and preventing liver fat accumulation, protects rats from hepatic insulin resistance [34]. Moreover, FFAs, which are associated with the development of liver steatosis, are inductors of insulin resistance via activation of protein kinases [35].

Indeed, it has been shown that hepatic protein kinase C (PKC) isoforms are involved in hepatocyte insulin resistance by inhibiting insulin signaling in human liver biopsy samples [36]. Samuel *et al*. showed that PKCɛ silencing by antisense oligonucleotide (ASO), leads to significant reduction in intra-hepatic triglycerides, hepatic insulin-resistance and fasting plasma insulin concentrations. PKCɛ silencing also restored insulin receptor substrate-2 (IRS-2) phosphorylation and protein-serine threonine kinase activity [37]. Activation of hepatic PKCɛ, was the best predictor of insulin resistance [38].

### 3.3. Lipid Intermediates Are Mediators of Insulin Resistance

Although triacylglycerol accumulation in the liver, as well as in the skeletal muscle, strongly correlates with insulin resistance, it is believed to be just a marker of increased levels of more potent disruptors of insulin signaling, such as fatty acid-derived lipids, which include lysophosphatidic acid (LPA), phosphatidic acid (PA) and diacilglycerol (DAG) [32]. These lipid intermediates and related molecules, such as acyl-CoA, ceramide, acyl-carnitines, activate several kinases, including protein kinase C (PKC), mTOR and S6K, which suppress IRS-1 tyrosine phosphorylation and downstream signaling [32,34,39,40]. There is evidence that hepatic TGs themselves are not toxic and may in fact protect the liver from lipotoxicity by buffering the accumulation of deleterious fatty acids [41]. Observations in mice with defects in various pathways that cause hepatic steatosis, but not insulin resistance, support this hypothesis [42–46]. Specific lipid species, the activation of specific pathways involved in fat accumulation, the cellular location and fat composition, the presence or absence of hepatic and systemic inflammation and the type of diet nutrients may all affect liver response to insulin [32]. While DAG, ceramide and high saturated fat diet are involved in hepatic insulin resistance, other lipid species variously affect hepatic insulin resistance. For example, glycerol-3-phosphate acyltransferase (GPAT1) knock-out mice are protected from hepatic insulin resistance when fed the high fat safflower oil diet, rich in DAG and ceramide, but not when fed a high saturated fat diet [47,48].

Yet, rats overexpressing ChREBP, fed on a standard diet, developed hepatic steatosis but remained insulin sensitive, despite increased expression of genes involved in lipogenesis/fatty acid esterification. Lipidomic analysis revealed that this model of steatosis was associated with increased accumulation of monounsaturated fatty acids (MUFAs), so that overexpression of ChREBP leads to modification in liver lipid composition [41].

Although these observations indicate that specific dietary lipids differently impair insulin signaling and action, additional studies are needed to support this notion.

## 4. An Emerging Role of NAFLD in Diabetes Prevention and Care

The liver plays a key role in regulating both glucose and lipid metabolism. Derangements in these metabolic pathways are typical features of both NAFLD and T2DM. In T2DM, fasting hyperglycemia results from unopposed endogenous hepatic glucose production, due to insulin resistance, and from postprandial hyperglycemia caused by reduced glucose uptake in skeletal muscle. Both fasting and postprandial hyperglycemia are, at least in part, linked to the amount of hepatic steatosis [49].

### 4.1. NAFLD: A New Predictive Marker of Diabetes

Diabetic patients have approximately 80% more fatty liver compared with non-diabetic controls matched for age and sex [50]. Interestingly, this association seems to be independent of glycemic control [51]. Indeed, NAFLD has been recently proposed as an independent risk factor for the future development of T2DM [52]. In addition, the development of diabetes is potentially associated with a more progressive NAFLD [53].

In the community-based Framingham Heart Study [54], enrolling 2589 individuals, fatty liver remained associated with T2DM, impaired fasting glucose, hypertension, metabolic syndrome, HDL cholesterol, triglycerides, and adiponectin levels, even after multivariate adjustment for other fat depots, such as visceral adipose tissue, waist circumference, and BMI.

A systematic meta-analysis [55] of 18 prospective, population-based studies (involving more than 70,000 subjects) showed that modestly increased serum GGT and ALT levels were independent and long-term predictors of T2DM. A larger meta-analysis [56] reported similar results. However, in this latter study, the results were adjusted only for some T2DM risk factors (age, sex, BMI/waist circumference, smoking, alcohol intake), but not for all other variables, including physical activity, family history of diabetes, fasting glucose, and insulin resistance.

In agreement with these data, a recent ultrasound survey of almost 3000 unselected patients with T2DM reported a NAFLD prevalence of 69.5% [57]. A meta-analysis published in 2011 [56] including three cohort studies in patients with NAFLD diagnosed by ultrasonography reported that NAFLD was associated with an increased risk of developing T2DM (adjusted odds ratio (OR), 3.51; 95% CI, 2.3–5.4). Further prospective cohort studies [58–62] reported that NAFLD was independently associated with an increased incidence of T2DM.

However, all these studies were performed in Asian populations, and the adjustment for potential confounders was often incomplete.

### 4.2. Fatty Liver and Diabetes: A Vicious Circle

Fatty liver is a major determinant in the development of T2DM in predisposed individuals [63]. However, once T2DM is fully developed, it further contributes not only to steatogenesis, but also to progressive liver damage including NASH, fibrosis, cirrhosis, and possibly HCC [64]. Patients simultaneously affected by T2DM and NAFLD often have poor glycemic control compared to their counterparts without NAFLD [65–67].

The intrahepatic triglyceride content is the major marker of the amount of insulin needed to achieve good glycemic control in T2DM patients. In fact, in insulin treated T2DM patients with stable glycemic control, the intrahepatic triglyceride content was the factor most strongly correlated with the daily insulin dose, the inter-individual variation in insulin requirements and with the ability of insulin to suppress hepatic glucose production [68] Many authors suggest that intrahepatic triglyceride content is more important than visceral fat content in inducing adverse metabolic phenotype in obesity [69–71]. Taken together, these data have identified NAFLD, not only as one of the more prominent chronic liver diseases, but also as a promising, new predictive marker of T2DM, with potential therapeutic implications. Diagnostic and early therapeutic interventions are needed for treating NAFLD patients at risk for developing T2DM. On the other hand, prevention or early diagnosis of progressive liver disease is needed in T2DM patients.

### 4.3. NAFLD Is Associated with Cardiovascular and Kidney Disease

A strong association between NAFLD and cardiovascular disease (CVD) has been long suspected. Several prospective, epidemiological studies [72–74] have suggested that NAFLD may represent an independent cardiovascular risk in addition to other risk factors such as dyslipidemia, diabetes and smoking.

Cross-sectional studies showed that NAFLD is strongly associated with increased carotid intima-media thickness and increased coronary artery calcium score [61,75–78]. NAFLD is also associated with early left ventricular diastolic dysfunction, decreased myocardial perfusion, and reduced myocardial high-energy phosphate metabolism in patients with T2DM [79–81]. In a large series of diabetic patients, it was reported [82] that NAFLD diagnosed with ultrasonography was associated with a significant increase in risk of incident CVD events after adjusting for several CVD risk factors.

NAFLD appears to be associated also with increased prevalence and incidence of chronic kidney disease (CKD) among patients with T2DM [52]. The Third National Health and Nutrition Examination Survey (NHANES-III) reported that an elevated serum γ-glutamyltransferase (GGT) level is independently associated with an increased prevalence of CKD [4]. Data from the Valpolicella Heart Diabetes Study [83] showed an almost double prevalence of CKD among T2DM patients with ultrasound-diagnosed NAFLD in comparison to those without it. Importantly, the same group reported that NAFLD was associated with an increased risk of incident CKD at follow-up in a cohort of 1800 diabetic patients, independently of other renal risk factors [84,85].

### 4.4. NAFLD, Diabetes and Cardiovascular Risk: A Molecular Link

Recent studies have been conducted to explain the putative mechanisms by which NAFLD increases T2DM risk, worsens glycemic control and contributes to the pathogenesis of major chronic complications of diabetes, such as CVD and CKD [52].

**Figure 1:**
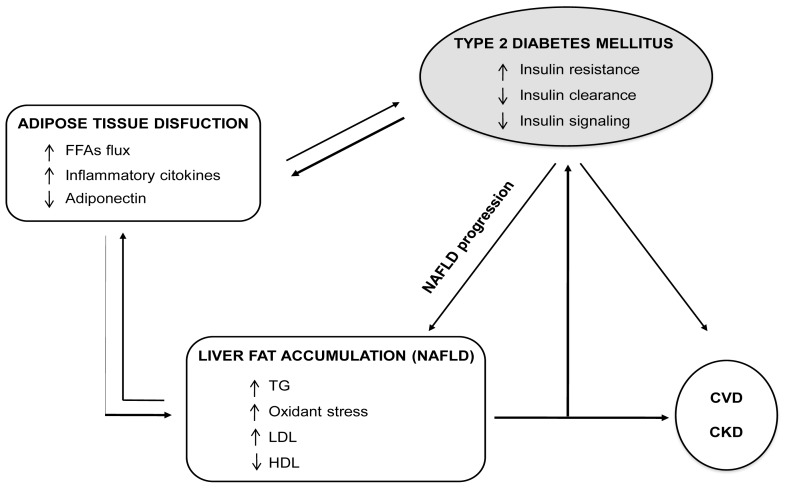
The pathogenetic link between non-alcoholic fatty liver disease (NAFLD), diabetes, CVD and CKD. Visceral adipose tissue releases inflammatory cytokines that induce liver damage. In turn, fatty liver is not only a target of these cytokines, but also the source of several proinflammatory, proatherogenic and nephrotoxic factors that may play a role in the development and progression of both cardiovascular disease (CVD) and chronic kidney disease (CKD). Moreover, NAFLD promotes T2DM development and enhances cardiovascular risk through the contribution to hepatic/systemic insulin resistance and atherogenic dyslipidemia. Additionally, NAFLD may contribute to the pathogenesis of T2DM through the release of some liver-secreted proteins with diabetogenic properties, such as fetuin-A, fibroblast growth factor-21, and retinol binding protein-4. On the other side, T2DM favors NAFLD progression.

In NAFLD patients, the liver is not only the target of inflammatory cytokines from visceral adipose tissue but also the source of several proatherogenic and nephrotoxic factors [52]. NAFLD, especially its necroinflammatory form, *i.e.*, the non-alcoholic steatohepatitis (NASH), may play a role in the development and progression of both CVD and CKD through the systemic release of several proinflammatory, procoagulant, and pro-oxidant mediators as well as through NAFLD mediated hepatic/systemic insulin resistance and atherogenic dyslipidemia. Additionally, NAFLD may contribute to the pathogenesis of T2DM through the release of some liver-secreted proteins with diabetogenic properties, such as fetuin-A, fibroblast growth factor-21, and retinol binding protein-4 [52]. The pathogenetic links between NAFLD, diabetes, CVD and CKD are summarized in Figure 1.

## 5. Diet and Physical Exercise in NAFLD Management

The clinical approach in NAFLD patients is primarily based on lifestyle changes, such as weight loss and physical exercise [3]. Most of the published studies in the NAFLD population have shown that gradual weight loss (5%–10%), calorie restricted diets and regular physical exercise may lead to improvement in liver enzyme profile and resolution of hepatic steatosis. In particular, weight reduction >5% was associated with improvement of steatosis and of cardio-metabolic risk factors, [86,87], while a reduction >7% may be associated with improvement of necro-inflammation [87–91]. However, most of these studies are non-randomized and short term. In contrast, a very fast weight loss (greater than 1.6 kg/week) may not be beneficial as it promotes massive mobilization of visceral fat, which goes directly to the liver via the portal vein [92]. Constant physical activity brings clear benefits to the metabolic abnormalities associated with NAFLD. The reduction of hepatic steatosis is due to the activation of protein kinases activated by adenosine monophosphate (AMPK). Physical exercise (aerobic) increases insulin sensitivity and improves the utilization of substrates in the muscles [93]. An optimal dietary treatment for NAFLD is not yet established. The majority of trials using a dietary treatment, according to the American Heart Association recommendations, include a caloric intake provided for the 40%–50% by carbohydrates, for 15%–20% by proteins and for 25%–40% by fatty acids, mainly unsaturated. A different nutrient composition of the diet may, however, provide additional benefits. Recent studies, in fact, demonstrate a role of cholesterol, saturated fatty acids and excess of fructose in the pathogenesis of NAFLD.

As NAFLD patients often show increased plasma triglycerides, hyperglycemia and hyperinsulinemia, they should follow a low carbohydrate diet with exclusion of simple sugars. Similarly, as these patients are often hypercholesterolemic, the diet must be hypolipidic with exclusion of saturated fat. The intake of vegetables and fruits should be strongly encouraged. Finally, the intake of alcoholic beverages should be strongly discouraged. The same applies to tobacco smoking, which should be avoided to reduce the cardiovascular risk associated with NAFLD [94]. Both weight loss and exercise significantly raise adiponectin and decrease leptin levels, improving insulin sensitivity in obese subjects. Adiponectin, in particular, is closely associated with insulin resistance in NAFLD [95].

However, the paucity of data prevents making evidence-based specific recommendations about dietary modifications and exercise in NAFLD patients. Moreover, dietary treatment is limited by the lack of compliance and the frequent weight regain at follow up.

## 6. Pharmacological Treatment of NAFLD

In spite of our increasing understanding of NAFLD pathogenesis, there is no consensus on an effective pharmacological treatment [96].

The clinical approach in NAFLD patients is primarily based on lifestyle changes, such as weight loss and physical exercise and is directed towards treating metabolic risk factors associated, such as diabetes, dislipidemia, hypertension [96]. However, many studies have evaluated specific pharmacological therapies, including insulin sensitizers (metformin and thiazolidinediones), weight loss drugs (orlistat and sibutramine), antioxidants (Vitamin E, sylimarin, betaine, pentoxifylline), and have also considered bariatric surgery for morbidly obese patients. Recently, the effects of the new antidiabetic drugs—DPP-4 inhibitors and glucagon-like peptide-1 (GLP-1) analogues—have been evaluated in diabetic NAFLD patients [97].

Other therapies currently undergoing evaluation in NASH include ACE inhibitors and angiotensin receptor blockers (ARBs) used for their antifibrotic properties. In fact, angiotensin has been shown to promote myofibroblasts survival and liver fibrosis [98].

Finally, as several cytokines as well as ER stress [99] are involved in NAFLD fibrogenesis, they may represent potential targets [100].

However, liver transplantation remains the only curative treatment option for end-stage cirrhosis. More studies are needed in this critical area, with particular focus on treatments, which can reverse or prevent the more advanced and clinically relevant stages of NASH.

## 7. NAFLD: A Possible New Target for Antidiabetic Drugs

As mentioned, NAFLD and diabetes share several pathogenetic mechanisms involving insulin signaling derangements, and are linked in a reciprocal cause–effect relationship. Accordingly, antidiabetics drugs, particularly insulin sensitizers, have been suggested to have a role in treating NAFLD and/or slowing down its progression. Our discussion focuses on the role of antidiabetic drugs in NAFLD therapy.

### 7.1. Metformin

Metformin is a biguanide widely used as the first-line therapy for patients with T2DM [101]. Metformin has beneficial effects on glucose and lipid metabolism. At molecular level, metformin inhibits the mitochondrial respiratory chain [102,103], inducing a transient reduction in cellular energy status that promotes the activation of adenosine monophosphate-activated protein kinase (AMPK), a key regulator of glucose and lipid metabolism. AMPK activation results in inhibition of hepatic gluconeogenesis and lipogenesis, increased glucose uptake in the muscle and increased fatty acid oxidation in the liver and adipose tissue. In adipose tissue, metformin also inhibits lipolysis and modulates adipokines synthesis and/or secretion [104].

#### 7.1.1. Preclinical Studies

Early evidence of metformin beneficial effects in hepatic steatosis comes from preclinical studies in insulin resistant mice with NAFLD [105]. Lin *et al*. first evaluated metformin treatment in insulin-resistant ob/ob mice with fatty liver. They found that metformin improved fatty liver disease, reversed hepatomegaly and steatosis, and normalyzed ALT levels [105].

Subsequently, in mice with methionine and choline-deficient, high fat diet (MCD + HF)-induced NAFLD [106], it was shown that metformin can prevent and reverse not only steatosis development but also liver inflammation.

#### 7.1.2. Clinical Studies

Recently, several clinical trials [107–115] have supported the beneficial role of metformin in NAFLD patients. These studies are somehow heterogeneous, as metformin has been used at various doses and patients included were diabetic or nondiabetic, and with various stages of NAFLD. Most of these studies have shown an improvement in liver biochemistry (aminotransferase levels) and metabolic syndrome features. However, to date, only few data are available regarding histological changes after metformin therapy.

In 2001, Marchesini *et al*. [109] conducted the first pilot study using metformin (1.5 g/day for 4 months) compared to diet in 20 non-diabetic NASH patients. They showed a significant decrease in insulin resistance, ALT levels, and improved liver morphology and volume in the metformin group. However, this study was non-randomized, conducted in a small group of patients, and liver histology was not evaluated.

The suggestion that metformin could have beneficial effects in liver histology of NASH patients comes from three open label single arm studies [108,110,113]. However, these studies are non-randomized and limited by the very small number of patients.

By contrast, four randomized trials comparing metformin plus lifestyle intervention with lifestyle intervention alone, for a period variable from six to 12 months, failed to observe a greater improvement in liver histology in the metformin group [111,114–116]. In the first study [111], 36 NASH patients were randomized to dietary treatment alone or dietary treatment plus metformin 1.7 g/day, for six months. The authors found no significant differences in necro-inflammatory activity or fibrosis between the two groups. However, serum aminotransferase and insulin resistance showed a greater improvement in the metformin group. A larger randomized study [114] involving 74 individuals with NASH showed similar results. Two subsequent studies [115,116] reported that metformin treatment did not improve either liver histology or biochemical parameters.

Two randomized trials evaluated the effects of metformin on radiological and biochemical indices in NAFLD patients at less advanced stages. In the first multicentric, randomized controlled trial, Bugianesi *et al*. [112] showed that metformin normalized aminotransferases in 69% cases *vs*. 31% of the diet group, and led to a greater amelioration in metabolic parameters. Limited histological data also suggested a beneficial effect of metformin in liver fat, necroinflammation and fibrosis. However, in a second randomized trial, Nar *et al*. did not observe a significant improvement of biochemical and radiological parameters when metformin was added to diet and exercise [117].

Recently, we conducted a prospective randomized study [107] in 50 obese, non-diabetic patients with early stage NAFLD. After six months, treatment with metformin (1 g/day) plus diet was associated with improvement of hepatic steatosis similar to that observed with diet treatment alone. However, the group treated with metformin plus diet showed significantly greater amelioration of insulin sensitivity and greater reduction of fasting glucose as compared to the diet group [107].

The largest trial using metformin in NAFLD was the TONIC study (Treatment of Nonalcoholic Liver Disease in Children) recently conducted in a pediatric population. It was a randomized, double-blind, placebo-controlled clinical trial conducted at 10 university clinical research centers involving 173 young patients (age 8–17 years) with biopsy-confirmed NAFLD, which were randomized to receive metformin (1 g/day), Vitamin E or placebo for 96 weeks. The study showed that metformin was not superior to placebo in reducing ALT levels and in ameliorating liver histology [118]. More details on randomized clinical trials using metformin in NAFLD patients are provided in Table 1.

A recent meta-analysis [119] concluded that, in NAFLD patients, metformin did not improve liver histology as compared with placebo, but significantly reduced body weight and insulin resistance. Overall, most of these studies were conducted in small series of patients, used different metformin doses and included various stages of NAFLD in diabetic or non-diabetic patients. Data on liver histology were limited by the small number of biopsies performed and the variable time periods between pre- and post-treatment biopsies.

Although clinical trials have yielded controversial results, they provide evidence that metformin, by improving the metabolic features of NAFLD, could be useful in the long term management of NAFLD patients. However, larger randomized controlled trials of sufficient duration and using histological endpoints are needed to fully assess the efficacy of this drug in modifying the natural history of NAFLD.

### 7.2. Thiazolidinediones

Thiazolidinediones (TZDs) are a class of insulin sensitizers, which include troglitazone, rosiglitazone and pioglitazone. The first two TZDs have been withdrawn from the market because of their significant side effects, whereas pioglitazone is currently available for clinic use in humans. TZDs act as agonists of the peroxisome-proliferator activated receptor gamma (PPARγ). PPARγ is a member of the nuclear receptor superfamily of ligand-activated transcription factors highly expressed in adipocytes. Its main function is to promote and maintain the whole body insulin sensitivity [123] by protecting non-adipose tissues against excessive lipid overload and by balancing the secretion of adipocytokines. Furthermore, TZDs also activate AMP-activated protein kinase and inhibit lipolysis, at least in part by inhibiting the translocation of hormone-sensitive lipase to lipid droplets [124].

#### 7.2.1. Preclinical Studies

Several mouse models have been used to elucidate the effectiveness of TZDs in NAFLD. In these studies, insulin-resistance and NAFLD have been induced by the administration of high fat diets or methionine-choline-deficient diets or by the genetic loss of leptin or leptin-receptor.

Early preclinical evidence of TZDs efficacy in NAFLD comes from studies conducted in animals treated with rosiglitazone. All these studies have shown that rosiglitazone treatment induces a clear morphological and molecular amelioration of hepatic steatosis [125,126]. Similar results have also been obtained by the use of pioglitazone [127], which improves not only insulin responsiveness but also the hepatic proliferative and regenerative response [128].

The molecular mechanisms proposed to explain the effectiveness of TZDs in these animal models of NAFLD are different [125,126,128,129]. However, it seems that all of them act in concert to increase fatty acid oxidation and to normalize the expression pattern of adipokines and subsequent cytokine responses.

#### 7.2.2. Clinical Trials

##### 7.2.2.1. Rosiglitazone

In humans, five studies—four open-label and one placebo-controlled trials—have evaluated the effect of rosiglitazone in NAFLD patients. The results of these studies are not uniform. Because rosiglitazone has been discontinued, we do not describe these studies, which are summarized in Table 2.

##### 7.2.2.2. Pioglitazone

Early pilot studies evaluating pioglitazone efficacy in the treatment of NAFLD reported a reduction of hepatic steatosis in the majority of treated patients. However, these promising and encouraging results were tempered by the small sample size and the lack of control placebo group [138].

Subsequently, larger controlled trials using pioglitazone showed greater promise. Belfort *et al*. published the first double-blind, placebo-controlled trial using diet plus pioglitazone (30–45 mg/day in 26 patients) and diet plus placebo (*n* = 21), both of which were compared to healthy controls (*n* = 10) for six months. The pioglitazone treated group showed an improvement in ALT (by 50%), steatosis (by 54%), insulin sensitivity (by 48%), liver inflammation and ballooning necrosis but not fibrosis [132]. In contrast to Belfort’s study, an improvement in fibrosis was seen in a similar trial conducted in 74 non-diabetic patients randomized to diet plus exercise, and either placebo or 30 mg/day of pioglitazone. The pioglitazone treated group (*n* = 31) revealed not only an improved fibrosis but also decreased liver enzymes levels and histological necro-inflammatory markers [133].

The largest multicenter placebo-controlled trial completed to date on the role of pioglitazone in 247 patients with biopsy-proven NASH and without diabetes and cirrhosis, is the PIVENS study (pioglitazone 30 mg/day, *n* = 80 *vs*. Vit E 800 IU/day, *n* = 84 and *vs*. placebo, *n* = 83; for 96 weeks). In this clinical trial, despite the pioglitazone group did not meet the primary endpoint (*i.e.*, improvement in histological findings assessed by the use of NAFLD Activity Score according to Kleiner *et al*. [139], pioglitazone use was associated with a significant amelioration of liver biochemistry, steatosis, liver inflammation, hepatocellular ballooning, as well as insulin resistance. Furthermore, 47% of patients receiving pioglitazone *vs*. 21% of placebo had complete resolution of steatohepatitis on end-of-treatment biopsy (*p* = 0.001) [137].

Two meta-analyses [140,141] evaluating some high-quality pioglitazone and rosiglitazone trials, concluded that TZDs improve histological steatosis and inflammation, but not fibrosis, compared with controls. In contrast to these results, a recent meta-analysis, evaluating four good quality clinical trials and excluding open label trials in which the control group received active treatment, have shown that TZDs, in particular pioglitazone, significantly improved all hepatic histological features, including fibrosis [142]. The discrepancies between these three meta-analyses may be due to the fact that in the latter study the authors conducted a subgroup analysis to assess the efficacy of pioglitazone alone.

However, independently of the effect on liver histology, the benefit-safety, long-term profile of TZDs, including pioglitazone, is not yet well established and warrants further assessment in larger trials of longer duration. Concerns regarding the long-term use of TZDs include weight gain, congestive heart failure, cardiovascular morbidity, bone loss and increased risk for urinary bladder cancer [143]. The development of selective PPARγ modulators (SPPARMS) could potentially open new hopes as they may have reduced side effects [144]. Clinical trials using TZDs in NAFLD patients are summarized in Table 2.

### 7.3. GLP-1 Analogs

Glucagon-like peptide-1 (GLP-1) and glucose-dependent insulinotropic polypeptide (GIP) are two peptides, named incretins, which are released by the gastrointestinal tract in response to nutrients, especially to glucose. Incretins’ main role is to amplify insulin secretion in a glucose-mediated manner [145]. Incretins also inhibit glucagon secretion, which in turn contributes to the inhibition of hepatic glucose production, [146] and have trophic and proliferative effects on beta cells [147]. GLP-1, but not GIP, inhibits the motility and secretion of the upper gastrointestinal tract, slows gastric emptying and suppresses appetite, thereby contributing to weight loss [148,149]. Importantly, GLP-1 also suppresses hepatic lipogenesis by activating the AMPK pathway, and reduces hepatic fat accumulation and nutrient-induced hepatic proinflammatory responses [150]. GLP-1 has a short half-life (1–2 min) due to rapid degradation by the enzyme dipeptidyl peptidase-4 (DPP-4). Two incretin-mimetics, exenatide and liraglutide, are currently used in T2DM therapy. These drugs are GLP-1 analogues resistant to DPP-4 degradation and with a longer half-life [151].

#### 7.3.1. Preclinical Studies

Ding *et al*. [152] first showed that exendin-4 treatment may induce the regression of hepatic steatosis and improve insulin sensitivity in ob/ob mice [152]. Subsequently, in high-fat fed mice, it was reported that GLP-1 receptor activation by exendin-4, inhibits VLDL production and reverses hepatic steatosis by decreasing hepatic lipogenesis [153]. In the animal model, GLP-1 analogues may also have beneficial effects in hepatic steatohepatitis, by reducing hepatic fat accumulation and inflammation [154,155].

Importantly, GLP-1 receptors are present in human hepatocytes and their activation produces a direct effect on hepatic steatosis, increasing hepatic insulin signaling and sensitivity [156]. Moreover, in human hepatocytes treated with fatty acids, exenatide reduced fatty acid accumulation and ER-stress mediated hepatocyte apoptosis [157].

#### 7.3.2. Clinical Studies

Only few studies are available on the clinical use of GLP-1 analogues in NAFLD patients. Most of them are non-randomized trials, and exclusively conducted in diabetic patients.

In a case report, the adjunct of exenatide therapy for 44 weeks to metformin monotherapy was shown to decrease hepatic fat accumulation, insulin resistance, and the risk of cardiovascular disease in a T2DM patient [158]. Similar results were reached in an open label clinical trial carried out in 217 diabetic patients over a period of three years [159]. These two studies, however, did not evaluate liver histology. The only study including pre- and post-treatment biopsies is a small, open label prospective study, conducted in eight diabetic NAFLD patients. Exenatide treatment improved serum ALT, but did not ameliorate liver histology [160].

The only data available on liraglutide come from a recent meta-analysis from six randomized clinical trials comprised in the “Liraglutide Effect and Action in Diabetes” (LEAD) program and including several thousand patients with NAFLD, treated with liraglutide (1.8 mg/day for 26 weeks). Treated patients showed a reduction in ALT and hepatic steatosis at computed tomography (CT) evaluation, as well as in NAFLD fibrosis score [161]. Taken together, these data support a beneficial role of GLP-1 analogues in human NAFLD. However, to date, randomized, placebo controlled clinical trials with NAFLD evaluation as primary endpoint, are lacking.

### 7.4. DPP-4 Inhibitors

DPP-4 inhibitors are used in T2DM treatment because of their ability to increase GLP-1 circulating half-life [162]. Several DPP-4 inhibitors, including alogliptin, saxagliptin, sitagliptin and vildagliptin are now available for clinical use [163]. DPP-4 is a ubiquitous enzyme that is expressed in all organs, including the small intestine, biliary tract, exocrine pancreas, spleen, and brain [162,164]. This widespread organ distribution indicates that DPP-4 has several biological activities, including regulatory effects on glucose metabolism, gut motility, appetite regulation, inflammation, immune and nervous system functions.

Liver expresses high DPP-4 levels, [162] which significantly increase in patients with NAFLD, compared with healthy subjects [165]. Moreover, serum DPP-4 activity and hepatic DPP-4 expression are correlated with NAFLD grading [166].

#### 7.4.1. Preclinical Studies

Recently, Shirakawa *et al*. [167] studied the effects of sitagliptin in Gck ± diabetic mice with diet-induced hepatic steatosis. Sitagliptin prevented fatty liver in both wild-type and Gck ± mice and decreased the expressions of sterol regulatory element-binding protein-1c, stearoyl-CoA desaturase-1, and fatty acid synthase, while it increased the expression of peroxisome proliferator-activated receptor-α in the liver. Further studies conducted in a mouse model of non-alcoholic steatohepatitis, indicated that linagliptin improves insulin sensitivity and hepatic steatosis in mice with diet-induced obesity [168] and may ameliorate liver inflammation [169].

#### 7.4.2. Clinical Studies

Clinical data are very limited, and come from non-randomized trials, all conducted in small groups of diabetic patients. A case report showed that a diabetic woman with refractory NAFLD was successfully treated with sitagliptin 50 mg/day. Glycemic control, ALT levels and liver fat, evaluated by Magnetic Resonance Imaging (MRI), improved after four months [163]. An open label single arm study [170] evaluated 30 NAFLD patients with T2DM treated with sitagliptin (50 mg/day) for four months. At the end of treatment AST, ALT and γ-GTP levels were reduced.

Moreover, another open-label single-arm study [171] recently evaluated the effectiveness of one-year treatment with sitagliptin (100 mg/day), on liver histology, body mass index (BMI), and laboratory parameters in 15 NASH patients with T2DM. Treated patients also showed a reduction in BMI, and ALT levels. Post-treatment liver biopsies showed a decrease in ballooning and NASH scores.

Overall, these studies suggest that DPP-4 inhibitors represent promising agents in NAFLD therapy, however, the paucity of data available does not allow for conclusions. A summary of clinical studies in T2MD/NAFLD patients employing GLP-1 analogues or DPP-4 inhibitors is given in Table 3.

## 8. Statins

Statins are cholesterol-lowering drugs that act trough the inhibition of 3 hydroxy-3 methyl-glutarylcoenzyme A reductase, the rate-limiting enzyme in cholesterol synthesis pathway. In addition to their beneficial effect on LDL cholesterol, statins also show several independent “pleiotropic” effects, mediated by the interference of statins with the mevalonate pathway. These effects include increased endothelial nitric oxide formation and flow-mediated vascular dilatation, suppression of vascular inflammation with decreased T-cell activation and cytokines production, and reduction of circulating inflammation biomarkers. Statins may also promote neovascularization in ischemic tissues, and modulate thrombosis and coagulation [172].

### 8.1. Preclinical Studies

Very recent preclinical studies have evaluated the effects of statins in animal models of NAFLD/NASH. In rats fed on a high-fat diet, Fraulob *et al*. [173] showed that rosuvastatin treatment improved insulin sensitivity and decreased liver steatosis. However, a similar study [174] found that, atorvastatin alone (30 mg/kg) for eight weeks led to only a moderate improvement of lipid profile and liver steatosis. Instead, dietary control alone or combined with atorvastatin treatment induced more significant improvement of hyperlipidemia and liver steatosis. Importantly, atorvastatin further improved the lipid profile, but did not enhance the beneficial effects of diet on hepatic steatosis. The effects on liver histology were not investigated.

However, other studies suggest that cholesterol-lowering drugs may improve liver histology in NASH rat models. In a recent study [175], ezetimibe (5 mg/kg) and atorvastatin (20 mg/kg) for eight weeks normalized hepatic free cholesterol, abolished JNK activation, improved serum ALT, apoptosis, liver inflammation/NAFLD activity score, and liver fibrosis. This effect was more marked using an ezetimibe/atorvastatin combination treatment. Another recent study [176] demonstrated that, in a high fat-induced NASH model, 12 week-treatment with rosuvastatin (2 mg/kg/day) improved not only hepatic steatosis but also hepatic injury and fibrosis via improved peroxisomal β-oxidation.

### 8.2. Clinical Studies

Beneficial effects of statins on liver enzymes, liver steatosis and inflammation have been shown in a number of small pilot studies[177,178].

However, only four randomized controlled trials (RCT) on statins’ efficacy in NAFDL patients are available [1,179–181]. Nelson *et al*. [180] evaluated the effects of simvastatin on liver histology. In this study, 16 biopsy-proven NASH patients were treated with simvastatin 40 mg for 12 months, but did not show an improvement in liver aminotransferases, liver histology and plasma lipids. However, only 14 subjects completed the study and only 10 had repeated liver biopsies. Thus, the small sample size may have contributed to the negative results.

The larger Greek Atorvastatin and Coronary Heart Disease study [1] was a three-year prospective, randomized, open label study, enrolling 1600 patients with established coronary heart disease, randomized to receive statins (atorvastatin 24 mg, simvastatin 22 mg, pravastatin 31 mg, fluvastatin 40 mg) or conventional therapy (lifestyle changes and treatment of associated risk factors). Primary endpoint was the first occurrence of any cardiovascular event. A post hoc analysis was performed in 437 patients (51% diabetics, 91% with metabolic syndrome) with NAFLD related abnormal baseline transaminases. Statin treatment, mainly with atorvastatin, was well tolerated and safe. Moreover, the incidence of new cases of diabetes was low (4%) and similar to that of patients not taking statins. After a three-year treatment, 89% of patients showed normalization of aminotransferase. By contrast, liver function tests worsened in NAFLD patients not receiving statins. NAFLD patients treated with statins also showed a decrease of LDL-cholesterol (−44% from baseline) and an increase of HDL-cholesterol (+32% from baseline). Importantly, statins treatment was associated with a significant risk reduction in cardiovascular events as compared with NAFLD patients not receiving statins.

The other large study was the St Francis Heart Study, [179] in which 1005 patients were enrolled and followed-up for 3.6 years. Patients included healthy individuals deemed high cardiovascular risk by coronary calcium score, which were randomized to receive atorvastatin (20 mg/day) plus Vitamin C (1 g/day) and Vitamin E (1000 IU/day), or placebo. In a subgroup of 455 subjects, abdominal computerized tomography was performed to identify patients with moderate-to-severe steatosis (>30%), and NAFLD was diagnosed in 80 patients. Patients with NASH, as identified by baseline transaminases exceeding 1.5 folds the upper normal value, were excluded. Only 59 patients completed the study, 27 of which were in the placebo group and 32 in the treated group. Patients in the treatment group showed not only a significant reduction in total cholesterol and LDL cholesterol levels but also a marked reduction of liver steatosis as compared with the placebo group. Significant reduction in liver steatosis was also observed in the small subgroup of normolipidemic NAFLD patients. This study, however, regarded only a subset of patients, and it could not dissect out the effect of atorvastatin from the effect of antioxidants.

Pramfalk *et al*. [181] confirmed the beneficial role of statins in 29 normo-cholesterolemic subjects. In this small, randomized clinical trial, atorvastatin (80 mg/day) reduced liver triacylglycerol content in biopsy samples by 40% compared to placebo. This effect was associated to changes in the expression of genes involved in hepatic lipogenesis.

A metanalysis [182] of these four trials concluded that statins may be of considerable benefit to NAFLD patients. Statins, especially atorvastatin, improved ultrasound and biochemical markers of liver disease and also reduced CVD events. However, their effects on liver histology are not yet elucidated. A summary of randomized clinical trials using statins in NAFLD patients is reported in Table 4.

Well-designed randomized controlled studies of adequate size and duration with histological endpoints are still needed in order to establish the beneficial effects of statins for hyperlipidemic and non-hyperlipidemic patients with NAFLD/NASH. A major issue for future studies would also be to identify predictors of response to statins in NAFLD patients, the efficacy of different statins and their effects on glucose metabolism.

## 9. Perspectives and Concluding Remarks

There is strong evidence that NAFLD is associated with increased risk of developing T2DM. NAFLD is also associated with a worse glycemic control and increased CVD/CKD risk in patients with established T2DM. These observations suggest that the occurrence of NAFLD should be taken into account in T2DM prevention and care. Patients with metabolic derangement should be screened for NAFLD, with the aim to reverse liver fat accumulation or to inhibit NAFLD progression, which in turn may help in preventing T2DM development. Similarly, patients with established T2DM should be also screened for NAFLD to avoid diabetes worsening and chronic complications. Conversely, a multidisciplinary approach is advocated in the treatment of patients with NAFLD, by evaluating cardiometabolic risk factors and monitoring for liver, cardiovascular and kidney complications.

In this view, an optimal pharmacological approach in T2DM patients should have a beneficial effect not only on the glycemic control but also on fatty liver, while drugs that worsen NAFLD should be avoided. Several studies suggest that antidiabetic drugs may have an important role in NAFLD therapy. Given the important role of insulin resistance in NAFLD development, insulin sensitizers have been mostly studied. Metformin treatment is safe and has been convincingly shown to improve the metabolic derangements associated with NAFLD. However, the few randomized studies to date available have not shown a significant improvement in liver histology. Pioglitazone appears to have beneficial effects on NASH histology; however, the available clinical data are limited by the small number of patients in which biopsies were performed. For both insulin sensitizers, studies on a large series of patients and with long-term follow-up are needed. The potential long-term adverse effects associated with the use of pioglitazone also need to be better addressed. The new antidiabetic drugs, DPP4-inibithors and GLP-1 analogues, may appear promising, but encouraging data mostly come from preclinical studies, and clinical trials are lacking.

Overall, further randomized trials are needed to confirm the beneficial effects of antidiabetic drugs, particularly on NAFLD histology. Finally, because most studies have been carried out in diabetic patients, new clinical trials must be conducted in non-diabetic patients with early-stage NAFLD, with the aim to investigate the ability of antidiabetic drugs in affecting not only liver steatosis but also T2DM incidence and cardiovascular risk.

## Figures and Tables

**Table 1:** Summary of clinical trials in NAFLD patients employing metformin.

References	Study design	Patients number and features	Therapy and duration follow-up	Study outcomes
Marchesini *et al*. 2001 [109]	OL, SA	20 pts (OB); NASH, elevated AMTs	Metformin 1.5 g/day; 4 months	↓ ALT ↓ IR ↓ Liver volume
Nair *et al*. 2004 [110]	OL, SA	28 pts (OW/OB/T2DM); NAFLD	Metformin 20 mg/kg/day; 12 months	↓ ALT, AST ↓ IR Histology improved
Uygun *et al*. 2004 [111]	OL, RAND	36 pts (OW/OB); NASH, elevated AMTs	Metformin 1.7 g/day + diet *vs*. diet; 6 months	↓ IR ↓ ALT, AST Histology not improved
Bugianesi *et al*. 2005 [112]	OL, RAND (MC)	110 pts (OW/OB/T2DM); NAFLD, elevated AMTs	Metformin 2 g/day + diet *vs*. Vit E + diet *vs*. diet; 12 months	↓ AST, ALT Histology improved
Schwimmer *et al*. 2005 [120]	SA	10 pts (OB/NT2DM children); NASH, elevated AMTs	Metformin 1 g/day; 6 months	↓ AST, ALT ↓ Liver fat ↓ IR
De Oliveira *et al*. 2008 [113]	OL, SA	20 pts (OW/OB/T2DM); NASH, elevated AMTs	Metformin 1 g/day; 12 months	↓ ALT ↓ IR Histology improved
Idilman *et al*. 2008 [114]	OL, RAND	74 pts (OW/OB/T2DM); NASH, elevated AMTs	Metformin 1.7 g/day; 12 months	↓ ALT ↓ IR Histology not improved
Nobili *et al*. 2008 [121]	OL	57 pts (OW/OB children); NASH/NAFLD	Metformin 1.5 g/day *vs*. diet; 24 months	↓ ALT, AST ↓ IR Histology improved
Shields *et al*. 2009 [116]	RAND, PLAC	19 pts (OW, OB, NT2DM); NASH, elevated AMTs	Metformin 1 g/day + lifestyle intervention *vs*. lifestyle intervention + placebo; 12 months	ALT, AST, IR and histology improved with BMI decrease (not significant, control *vs*. treatment)
Loomba *et al*. 2009 [108]	OL, SA	28 pts (OW/OB/T2DM); NASH, elevated AMTs	Metformin 2 g/day; 12 months	↓ ALT, AST ↓ IR Histology improved
Haukeland *et al*. 2009 [115]	PLAC, RAND	48 pts (OW/OB/T2DM); NAFLD, elevated AMTs	Metformin *vs*. placebo; 6 months	↓ ALT, AST ↓ IR Histology not improved
Nadeau *et al*. 2009 [122]	RAND	50 pts (OB children); NAFLD, elevated AMTs	Metformin 1.7 g/day + diet *vs*. diet; 6 months	↓ ALT, AST ↓ IR Ultrasound pattern improved
Garinis *et al*. 2010 [107]	OL, RAND	50 pts (OW/OB); NAFLD, normal AMTs	Metformin 1 g/day + diet *vs*. diet; 6 months	↓ ALT, AST ↓ IR Ultrasound pattern improved ↑ Adiponectin
Lavine *et al*. 2010 [118]	RAND (MC)	173 pts (OW/OB children); NAFLD, elevated AMTs	Metformin 1 g/day *vs*. Vit E; 24 months	Not stable AST e ALT reduction Histology not improved

Abbreviations: ALT, alanine transaminase; AMTs, aminotransferases; AST, aspartate aminotransferase; IR, insulin resistance; NAFLD, nonalcoholic fatty liver disease; NASH, nonalcoholic steatohepatitis; MC, multicentric; NT2DM, non type 2 diabetes mellitus; OB, obese; OL, open label; OW, overweight; PLAC, placebo controlled; RAND, randomized; SA, single arm; T2DM, type 2 diabetes mellitus.

**Table 2:** Summary of clinical trials in NAFLD patients employing thiazolidinediones (TZDs).

References	Study design	Patients number and features	Therapy and duration follow-up	Study outcomes
Wang *et al*. 2006 [130]	OL	60 pts (DM2); NAFLD, elevated AMTs	Rosiglitazone 4–8 mg/day; 24 weeks	↓ AMTs ↓ IR ↓ fasting plasma glucose and insulin ↓ HBA1C
Akyuz *et al*. 2007 [131]	OL	11 pts NAFLD, elevated ALT	Rosiglitazone 4 mg/day *vs*. metformin *vs*. diet; 12 months	↓ AMTs ↓ IR Histology not improved
Idilman *et al*. 2008 [114]	OL, RAND	25 pts; NASH, elevated AMTs	Rosiglitazone 8 mg/day *vs*. metformin *vs*. diet; 12 months	↓ AMTs Histology improved ↓ IR ↓ C-reactive protein
Belfort *et al*. 2006 [132]	PLAC, RAND	26 pts (IGT/DM2); NASH	Pioglitazone 30 mg/day *vs*. placebo for the first 2 months, then 45 mg/day for 4 more months	↓ ALT ↓ IR ↓ Liver fat ↓ Liver inflammation ↓ Ballooning necrosis Fibrosis not improved
Aithal *et al*. 2008 [133]	PLAC, RAND	31 pts; NASH	Pioglitazone 30 mg/day *vs*. placebo; 12 months	↓ ALT improved ↓ Liver inflammation Fibrosis improved
Ratziu V *et al*. 2008 [134]	OL, PLAC, RAND	32 pts; NASH, elevated AMTs	Rosiglitazone 4 mg/day *vs*. placebo for the first month then 8 mg/day for 11 more months	↓ AMTS ↓ Liver fat ↓ IR
Omer *et al*. 2010 [135]	OL, RAND	42 pts; (IGT/DM2); NAFLD, elevated ALT	Rosiglitazone 4 mg/day alone *vs*. Rosiglitazone + metformin; 12 months	↓ ALT ↓ IR ↓ Liver fat Fibrosis not improved
Ratziu V *et al*. 2010 [136]	OL extension, RAND	18 pts; NASH	Rosiglitazone 8 mg/day, 3 years	↓ ALT ↓ IR Histology not further improved
Sanyal AJ *et al*. 2010 [137]	PLAC, RAND	80 pts; NASH	Pioglitazone 30 mg/day *vs*. Vit E *vs*. placebo; 96 weeks	↓ ALT ↓ IR ↓ Liver fat ↓ Liver inflammation ↓ Ballooning necrosis Fibrosis not improved

Abbreviations: ALT, alanine transaminase; AMTs, aminotransferases; AST, aspartate aminotransferase; IGT, impaired fasting glucose; IR, insulin resistance; NAFLD, nonalcoholic fatty liver disease; NASH, nonalcoholic steatohepatitis; NT2DM, non type 2 diabetes mellitus; OB, obese; OL, open label; OW, overweight; PLAC, placebo controlled; RAND, randomized; SA, single arm; T2DM, type 2 diabetes mellitus.

**Table 3:** Summary of clinical trials in T2MD/NAFLD patients employing GLP-1 analogues or DPP-4 inhibitors.

References	Study design	Patients number and features	Therapy and duration follow-up	Study outcomes
Tushuizen *et al*. 2006 [158]	Case report	1 pt (T2DM/NASH), elevated AMTs	Exenatide 10 μg plus metformin; 9 months	↓ ALT, AST ↓ Liver fat (MRS) ↓ IR and CVD risk factors Histology not assessed
Klonoff *et al*. 2008 [159]	OL	217 pts (OW/OB/T2DM); NAFLD elevated/normal AMTs	Exenatide 10 μg plus metformin and/or sulfanilureas; 36 months	↓ ALT, AST ↓ IR Histology not assessed
Kenny *et al*. 2010 [160]	Case series	8 pts (T2DM); NAFLD, elevated AMTs	Exenatide 10 μg; 6 months	↓ AST, ALT Histology not improved
Iwasaki *et al*. 2011 [170]	OL, SA	30 pts (T2DM); NAFLD elevated/normal AMTs	Sitagliptin 50 mg; 4 months	↓ ALT, AST, GGT Histology not assessed
Itou *et al*. 2012 [163]	Case report	1 pt (T2MD); NAFLD elevated AMTs	Sitagliptin 50 mg; 4 months	↓ AST, ALT ↓ IR ↓ Liver fat (MRI) Histology not assessed
Ylmaz *et al*. 2012 [171]	OL, SA	15 pts (T2DM); NASH elevated AMTs	Sitagliptin 100 mg; 12 months	↓ ALT, AST Histology improved
Armstrong *et al*. 2010 [161]	RAND, PLAC, MC (meta-analys of 6 studies + sub-study)	4442 pts (T2DM); NAFLD elevated/normal AMTs	Liraglutide 1.8 mg *vs*. OAD or placebo; 6 months	↓ ALT ↓ Liver fat (CT) Histology not assessed

Abbreviations: ALT, alanine transaminase; AMTs, aminotransferases; AST, aspartate aminotransferase; CT, computerized tomography; IR, insulin resistance; NAFLD, nonalcoholic fatty liver disease; NASH, nonalcoholic steatohepatitis; OB, obese; OW, overweight; PLAC, placebo controlled; RAND, randomized; T2DM, type 2 diabetes mellitus.

**Table 4:** Summary of clinical trials in NAFLD patients employing statins.

References	Study design	Patients number and features	Therapy and duration follow up	Study outcomes
Nelson *et al*. 2009 [180]	RAND PLAC	16 pts NASH, elevated AMTs	Simvastatin 40 g/day; 12 months	AST, ALT not reduced Histology not improved
Athyros *et al*. 2010 [1]	RAND PLAC	437 pts (OB/44%T2DM); NAFLD, elevated AMTs	Atorvastatin 24 mg/day or other statins; 36 months	↓ AST, ALT ↓ Total cholesterol, triglycerides Histology not assessed
Foster *et al*. 2011 [179]	RAND PLAC	80 pts (OB/51%T2DM) NAFLD	Atorvastatin 20 mg/day + Vit C + Vit E; 42 months	AST, ALT not reduced ↓ Liver fat (CT) ↓ Total cholesterol, LDL Histology not assessed
Pramfalk *et al*. 2011 [181]	RAND PLAC	19 pts NAFLD, normocholesterolemic	Atorvastatin 80 mg/day; 1 month	AST, ALT not assesed ↓ Liver fat (biopsy) ↓ lipogenic genes expression IR not reduced

Abbreviations: ALT, alanine transaminase; AMTs, aminotransferases; AST, aspartate aminotransferase; CT, computerized tomography; IR, insulin resistance; NAFLD, nonalcoholic fatty liver disease; NASH, nonalcoholic steatohepatitis; OB, obese; OW, overweight; PLAC, placebo controlled; RAND, randomized; T2DM, type 2 diabetes mellitus.
